# Plant‐based expression and characterization of SARS‐CoV‐2 virus‐like particles presenting a native spike protein

**DOI:** 10.1111/pbi.13813

**Published:** 2022-04-06

**Authors:** Jae‐Wan Jung, Gergana Zahmanova, Ivan Minkov, George P. Lomonossoff

**Affiliations:** ^1^ Department of Biochemistry and Metabolism John Innes Centre Norwich Research Park Norwich UK; ^2^ 26714 Department of Molecular Biology Jeonbuk National University Jeonju Korea; ^3^ Department of Plant Physiology and Molecular Biology University of Plovdiv Plovdiv Bulgaria; ^4^ 585099 Center of Plant Systems Biology and Biotechnology Plovdiv Bulgaria; ^5^ 585099 Institute of Molecular Biology and Biotechnologies Markovo Bulgaria

**Keywords:** severe acute respiratory syndrome coronavirus 2, virus‐like particles, spike protein, COVID‐19, *Nicotiana benthamiana*, transient expression

## Abstract

We have investigated the use of transient expression to produce virus‐like particles (VLPs) of severe acute respiratory syndrome coronavirus 2, the causative agent of COVID‐19, in *Nicotiana benthamiana*. Expression of a native form of the spike (S) protein, either alone or in combination with the envelope (E) and membrane (M) proteins, all of which were directed to the plant membranes via their native sequences, was assessed. The full‐length S protein, together with degradation products, could be detected in total protein extracts from infiltrated leaves in both cases. Particles with a characteristic ‘crown‐shaped’ or ‘spiky’ structure could be purified by density gradient centrifugation. Enzyme‐linked immunosorbent assays using anti‐S antibodies showed that threefold higher levels of VLPs containing the full‐length S protein were obtained by infiltration with S alone, compared to co‐infiltration of S with M and E. The S protein within the VLPs could be cleaved by furin *in vitro* and the particles showed reactivity with serum from recovering COVID‐19 patients, but not with human serum taken before the pandemic. These studies show that the native S protein expressed in plants has biological properties similar to those of the parent virus. We show that the approach undertaken is suitable for the production of VLPs from emerging strains and we anticipate that the material will be suitable for functional studies of the S protein, including the assessment of the effects of specific mutations. As the plant‐made material is noninfectious, it does not have to be handled under conditions of high containment.

## Introduction

The ongoing COVID‐19 pandemic, caused by severe acute respiratory syndrome coronavirus 2 (SARS‐CoV‐2), has led to a serious health crisis with an ever‐increasing number of cases. As of September 2021, more than 217 million cases and at least 4.5 million deaths have been reported worldwide. In common with other human coronaviruses, SARS‐CoV‐2 causes respiratory disease, associated with high fever, difficulty in breathing, and pneumonia. However, SARS‐CoV‐2 infections are sometimes asymptomatic or result in only very mild symptoms, leading to the rapid spread of the virus (Chen *et al*., 2020).

SARS‐CoV‐2 is an enveloped positive‐strand RNA virus and is a member of the *Betacoronavirus* genus, subgenus *Sarbecovirus* (Pal *et al*., 2020). Genus *Betacoronavirus* also harbours the highly pathogenic SARS‐CoV (first identified in 2003), Middle East respiratory syndrome coronavirus (MERS‐CoV), reported in 2013, and the so‐called ‘common cold’ human coronaviruses, hCoV‐OC43, and hCoV‐HKU (Liu *et al*., 2021). *Betacoronavirus* particles are enveloped and are spherical or pleomorphic in shape, with an average diameter of 80–120 nm. The coronavirus genome is composed of a conserved region encoding an RNA‐dependent RNA polymerase and a variable region containing open reading frames that encode a number of viral proteins, including the spike (S), envelope (E), and membrane (M) proteins that are associated with the host‐encoded lipid membrane (Lu *et al*., 2020; Ying *et al*., 2004).

The S protein of coronaviruses plays a major role in virus uptake into target cells and can elicit effective immune responses in mammals after vaccination (Gomez *et al*., 2000; Huy *et al*., 2016; Li *et al*., 2006; Walsh *et al*., 2020). The S protein is a type I transmembrane (TM) protein, with a molecular mass of 128–160 kDa before glycosylation and 150–200 kDa after N‐linked glycosylation. The S glycoprotein contains an N‐terminal ectodomain, a single TM domain and a short C‐terminal endodomain and forms homotrimer structures that are anchored to the virus envelope via the TM domain (Shen *et al*., 2004). Each S protein monomer consists of a globular N‐terminal region (S1), representing the outer part of the virus, with the C‐terminal S2 domain forming a stalk that contains the TM domain (Fung and Liu, 2018; Letko *et al*., 2020; Walls *et al*., 2020; Yamada and Liu, 2009). The S1 and S2 domains are bounded by a furin cleavage site (Vankadari, 2020). SARS‐CoV‐2 binds to target cells through an interaction between the receptor‐binding domain on S1 and human angiotensin‐converting enzyme 2 on susceptible cells. Following activation by furin cleavage, the exposed S2 domain mediates membrane fusion (Huang *et al*., 2020; Shang *et al*., 2020). The signal peptide at the N‐terminus of the S protein, and its ER retrieval signal (‘KxHxx’ motif) in the C‐terminal TM domain, leads to its subcellular localization on the host membranes (Hu *et al*., 2020).

E is a small (8–12 kDa) integral membrane protein, found in limited amounts in the virion, but likely to play an important role in virus assembly (Fung and Liu, 2018). The most abundant structural protein, M, interacts with all other structural proteins (S, E, and N) (Masters, 2006; Neuman *et al*., 2011; Siu *et al*., 2008). A strong interaction between E and M protein results in co‐localization and co‐translocation to the same subcellular compartments when they are expressed together (Corse and Machamer, 2003; Lim and Liu, 2001; Park *et al*., 2021).

Given the severity of the pandemic caused by SARS‐CoV‐2, there have been intensive efforts to develop effective vaccines against the virus. Most of these efforts have focused on the S protein or portions thereof (Martinez‐Flores *et al*., 2021). Currently, lipid nanoparticle‐formulated, nucleoside‐modified RNA vaccines (Polack *et al*., 2020; Walsh *et al*., 2020) and adenovirus‐based vaccines that express the S protein within cells have been widely deployed to elicit immunity. In addition, vaccines based on soluble S protein subunits (Tian *et al*., 2021), or virus‐like particles (VLPs) (Ward *et al*., 2021) are also under development. VLPs are highly similar to native viral particles in structure and antigenicity but lack the viral genome and are hence noninfectious (Bachmann and Jennings, 2010). Thus, VLPs are attractive candidates for vaccine development and are widely used in this role. VLPs of coronaviruses of both veterinary and medical importance, including SARS‐CoV‐2, have been successfully produced in insect or mammalian cells by co‐expressing the E, M, and S proteins (Bai *et al*., 2008; Lokugamage *et al*., 2008; Lu *et al*., 2007, 2010). Plant‐based expression (Ward *et al*., 2021) has recently been used to express a version of the S protein modified to contain point mutations to stabilize it in the prefusion form (Wrapp *et al*., 2020). In addition, the native signal sequence was replaced with a plant signal sequence and the TM domain and cytoplasmic tail of S were replaced with the equivalent sequences from influenza haemagglutinin. Clinical trials phases 2–3 with these VLPs are currently ongoing (Ward *et al*., 2021).

Because of the containment requirements for handling infectious SARS‐CoV‐2, there is currently a need for noninfectious surrogates to enable studies on such aspects as cell binding to be carried out under less stringent conditions. Such surrogates should be as similar as possible to the native virus structure. We have recently obtained preliminary evidence that VLPs of porcine epidemic diarrhoea virus and SARS‐CoV‐2 could be produced in plants using native S protein sequences (Peyret *et al*., 2021). To further assess the requirements for successful production of SARS‐CoV‐2 VLPs, we have now transiently expressed a native version of the S protein, either alone or in combination with the M and E proteins, in *Nicotiana benthamiana* and report the characterization of the resulting VLPs.

## Results

### Agroinfiltration and protein expression

**Figure 1:**
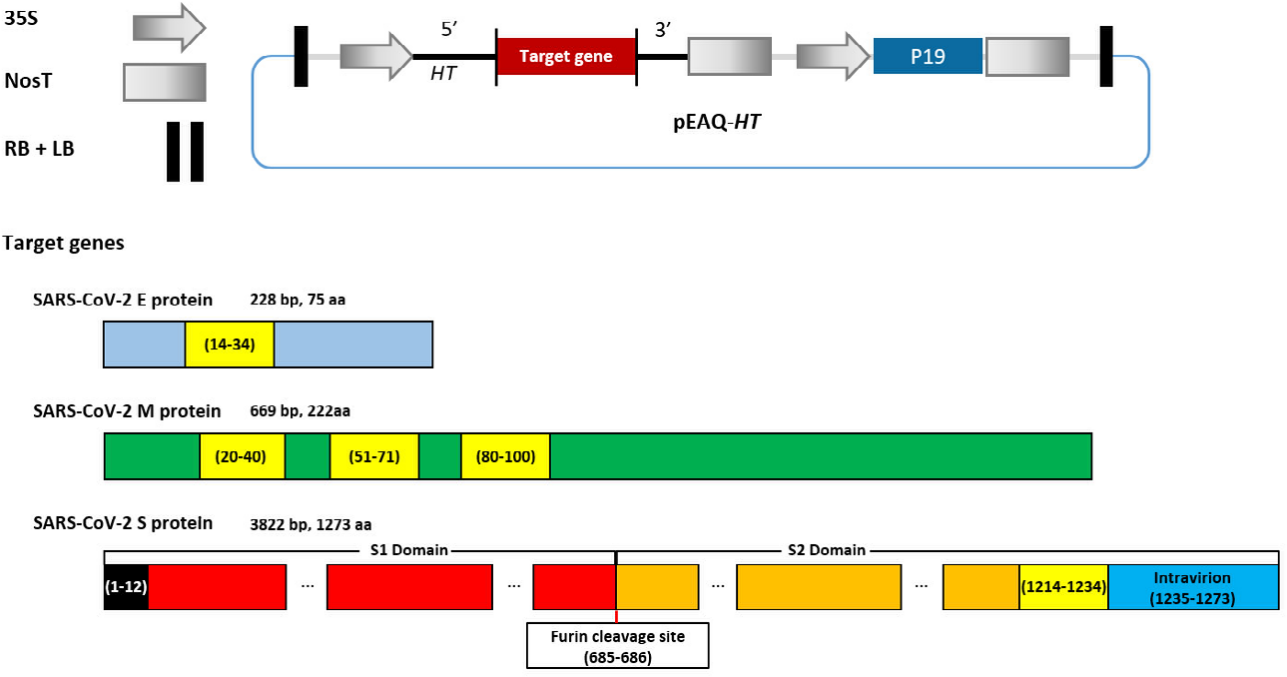
Vector construction for expression of SARS‐CoV‐2 structural proteins, E, M, and S. LB: T‐DNA left border; RB: T‐DNA right border; 35S: 35S promoter of cauliflower mosaic virus; 5′: 5′‐UTR from cowpea mosaic virus RNA‐2; 3′: 3′‐UTR from cowpea mosaic virus RNA‐2; NosT: nopaline synthase terminator; P19: suppressor of silencing; the numbers in parentheses refer to the amino acid sequence. Yellow box: TM domain and black box: signal peptide sequence.

*Agrobacterium tumefaciens* suspensions harbouring plasmids, pEAQ‐*HT*‐E, pEAQ‐*HT*‐M, or pEAQ‐*HT*‐S (Figure 1), designed to express native versions of the SARS‐CoV‐2 E, M, and S protein, respectively, were infiltrated into *N. benthamiana* leaves either individually or in combination. Leaves infiltrated with pEAQ‐*HT*‐M alone (M) showed necrosis by 4 dpi while those infiltrated with pEAQ‐*HT*‐E (E), pEAQ‐*HT*‐S (S), or a combination of all three plasmids (EMS) showed only mild chlorosis similar to that observed in leaves infiltrated with the empty vector (EV) at 6 dpi. Thus, co‐expression of E and S with M seems to alleviate the necrosis associated with infiltration with M alone (Figure S1a).

**Figure 2:**
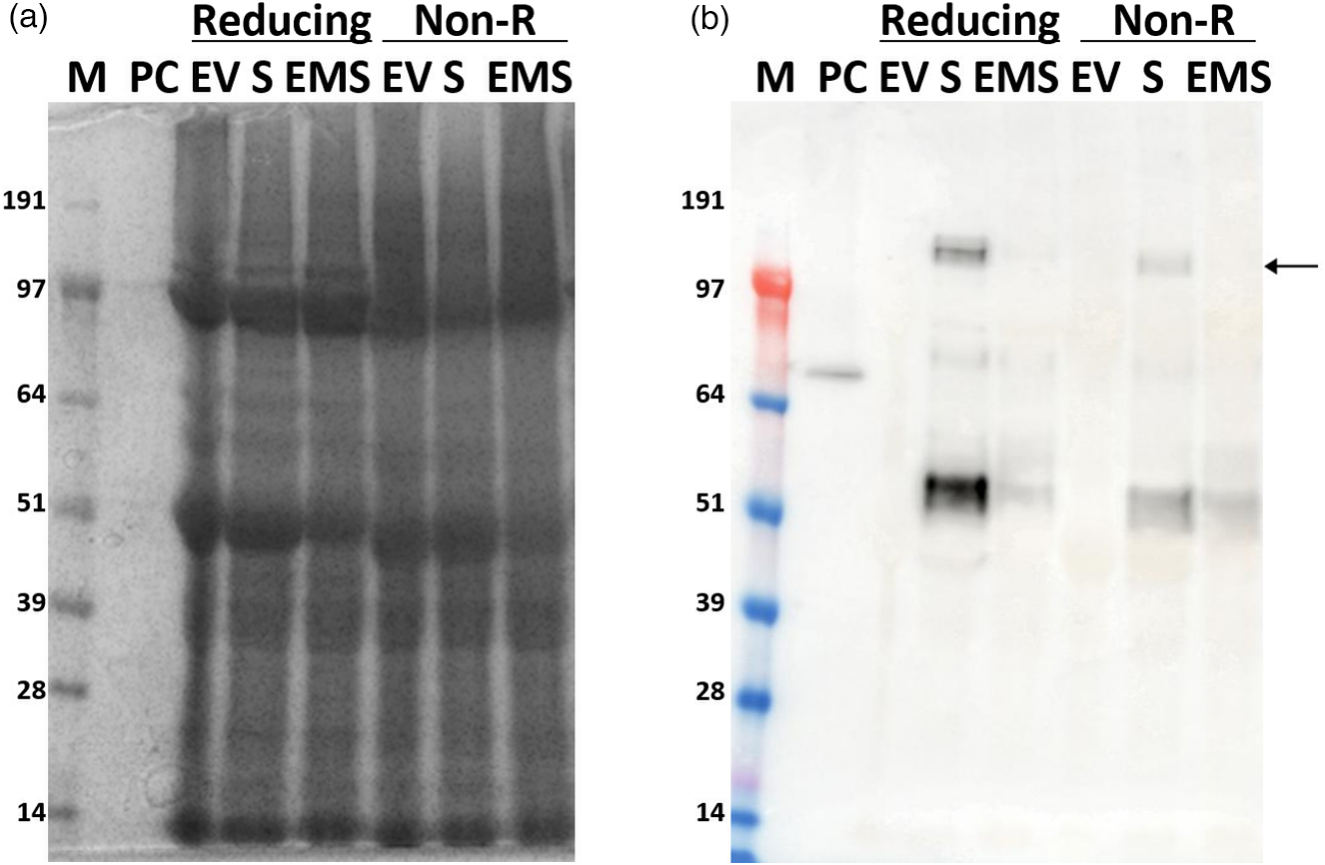
Analysis of crude extracts of leaves infiltrated with S or EMS constructs. Protein extracts from 6 dpi leaves infiltrated with empty vector (EV), S, or EMS were analysed by SDS–PAGE (a) and Western blot using anti‐SARS‐CoV‐2 S protein antibody (b). Reducing: samples boiled in the presence of β‐mercaptoethanol; Non‐R: samples boiled in the absence of β‐mercaptoethanol; PC, positive control, 50 ng of SARS‐CoV‐2 S‐E‐M mosaic protein with His‐tag. The arrow indicates the monomeric form of the full‐length S protein. Lane M, protein size markers.

To determine whether expression of SARS‐CoV‐2 S protein occurred in infiltrated leaves, total protein extracts were prepared from leaves infiltrated with pEAQ‐*HT*‐S (S) alone or a combination of all three plasmids (EMS) and analysed by sodium dodecyl sulphate–polyacrylamide gel electrophoresis (SDS–PAGE) under both reducing and nonreducing conditions. Western blot analysis using a polyclonal anti‐S protein antibody showed the presence of a band of over 100 kDa, consistent with the presence of full‐length S protein, as well as a strong band of approximately 55 kDa which presumably represents a cleavage product of the S protein, in both the S and EMS samples irrespective of whether reducing or nonreducing conditions were used (Figure 2). The use of alternative buffers, such as phosphate‐buffered saline (PBS), and the addition of cOmplete protease inhibitor cocktail (Roche Diagnostics GmbH, Mannhein, Germany) made no detectable difference to the pattern. It therefore seems probable that the observed degradation occurs prior to extraction. The intensity of the signals was consistently higher in the case of infiltration with S alone, indicating that the presence of the E and M proteins reduces the level of accumulation of the protein. This is supported by the observation that infiltration with M protein alone causes considerable damage to the leaves. The effect of *Agrobacterium* concentration on S protein accumulation was also examined and showed that increasing the concentration to OD_600_ = 0.9 or 0.6 was deleterious. (Figure S1b,c). This appears to be related to the severity of the symptoms observed on infiltrated leaves.

**Figure 3:**
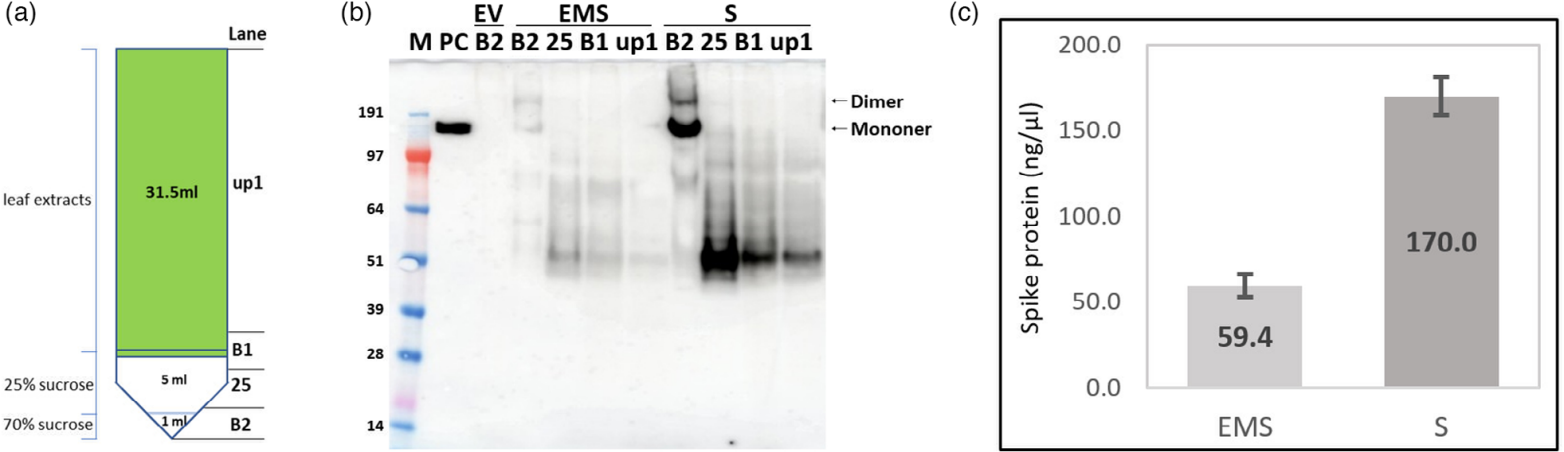
Detection of SARS‐CoV‐2 S protein in fractions during purification. (a) Scheme of purification using a double sucrose cushion. Up1 represents the material that did not penetrate the cushions, B1 the interface between the upper layer and the 25% (w/v) sucrose layer, 25 the 25% (w/v) sucrose fraction, and B2 the interface between the 25% and 70% sucrose layers plus the 70% sucrose layer. (b) Each fraction from the sucrose cushion was collected and analysed by Western blot using anti‐SARS‐CoV‐2 S protein antibody. Lane M, Protein marker; Lane PC, 50 ng of SARS‐CoV‐2 Spike protein from CHO cell. (c) Levels of S protein in VLP samples after purification over iodixanol gradients as measured by ELISA. The values are presented after subtraction of the negative control values from extracts from EV‐infiltrated leaves. Error bars indicate the standard deviation of averages from three independent experiments.

To assess whether the expressed S protein was incorporated into higher‐order structures, such as VLPs, extracts from leaves infiltrated with S or EMS were analysed by ultracentrifugation through a double sucrose cushion (Figure 3a; Peyret, 2015). Western blot analysis of the various fractions showed that the full‐length S protein was predominately associated with the material collecting at the interface between 25% and 70% sucrose (B2) layer, while the lower molecular mass products were found in the upper, less dense, fractions (Figure 3b). This distribution was the same for both the S and EMS samples, though, as noted above, the intensity of the signals was greater in the preparations from leaves infiltrated with S alone. In addition to the full‐length S protein, higher molecular mass forms of the S protein, including dimers, could also be detected in fraction B2. As these were not found at such levels in samples analysed immediately after extraction (Figure 2b), they are probably formed by cross‐linking between adjacent S protein monomers as a result of the extended exposure to plant sap during VLP purification as has previously reported (Castells‐Graells and Lomonossoff, 2021). The origin of the lower molecular mass bands was investigated by comparing Western blots probed with polyclonal antibodies raised against either full‐length S protein or just the S2 domain. The results (Figure S2) indicate that this material contains S protein fragments from both the S1 and S2 domains. Overall, the data indicate that full‐length S protein, but not its cleavage products, are present in higher‐order structures such as VLPs.

### Extraction and characterization of plant‐produced SARS‐CoV‐2 VLPs

To further purify potential plant‐produced SARS‐CoV‐2 VLPs, the B2 fraction from the double sucrose cushions of the S and EMS preparations (Figure 3) was desalted and centrifuged through 12%–30% (w/v) iodixanol step gradients, and the fractions analysed by Western blotting using the anti‐SARS‐CoV‐2 S protein antibody (Figure S3). In each case, fraction #6 (the interface between 18% and 24% (w/v) iodixanol) showed the highest level of full‐length S protein; thus, this fraction was used for further analysis. EV material was also prepared and fractionated using the same conditions and used as a negative control. Indirect enzyme‐linked immunosorbent assays (ELISA) was conducted (Figure 3c), using commercial Chinese hamster ovary (CHO) cell‐expressed trimeric S protein to produce the standard curve, and showed that the concentration of purified S protein from leaves infiltrated with S alone or EMS were 170.0 ± 11.3 and 59.4 ± 6.8 ng/μL, respectively; the EV sample showed negligible levels. These levels equate to overall yields of approximatively 23 and 8 mg of full‐length S protein per Kg wet mass of infiltrated leaves. The approximately threefold difference in yield between the S and EMS samples is consistent with the previous Western blot analysis (Figure 3b). This was further confirmed by comparing the amounts of full‐length S protein in each sample with known amounts of commercially available S protein on a stained gel (Figure S3h).

### Observation of VLPs by electron microscopy

**Figure 4:**
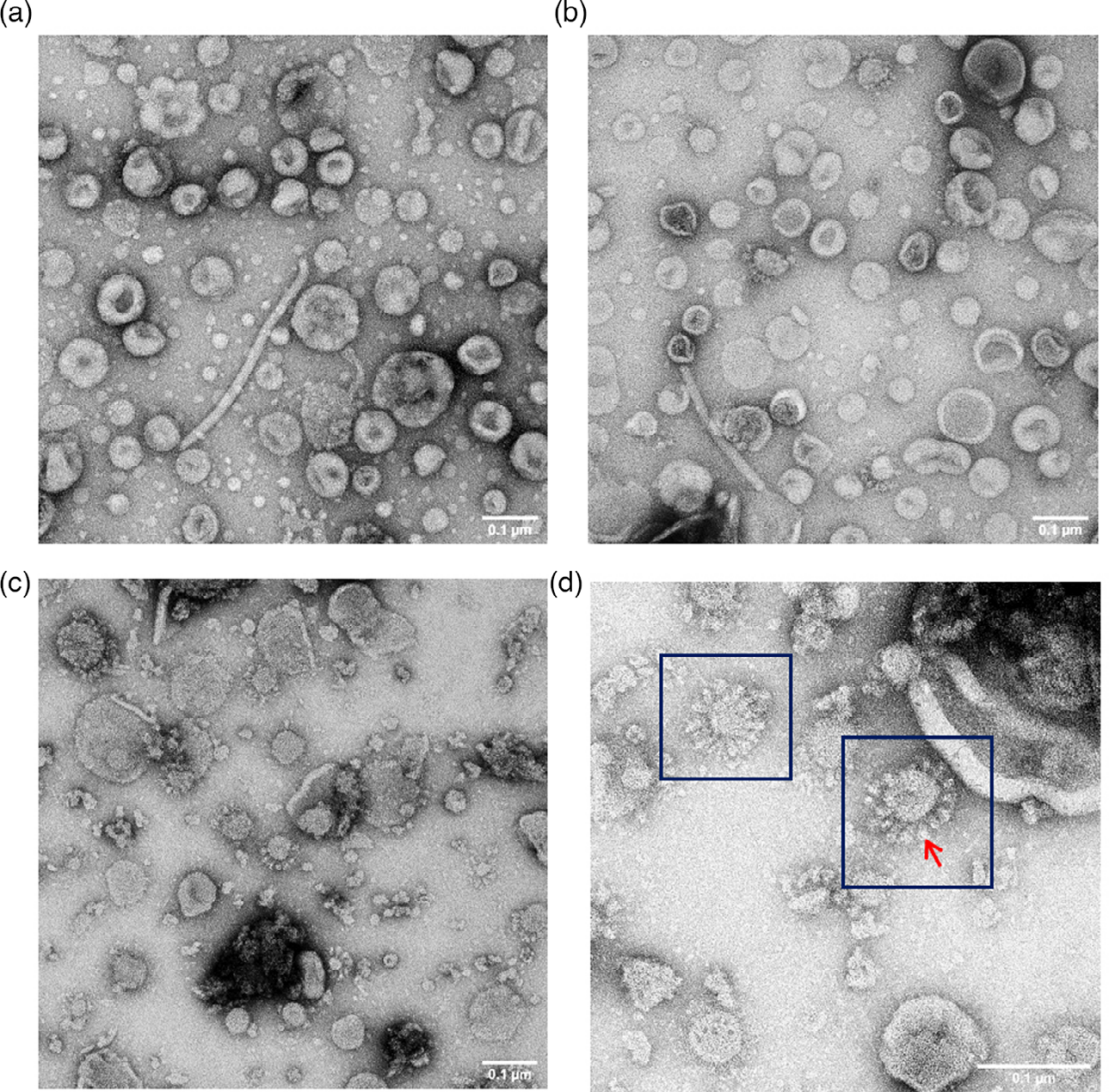
Observation of SARS‐CoV‐2 VLPs using transmission electron microscopy. (a) Purified fraction #6 from EV‐infiltrated leaves prepared by the same method as EMS and S. (b) Fraction #6 from leaves infiltrated with EMS. (c) Fraction #6 from leaves infiltrated with S. (d) image of VLPs in S sample at higher magnification (150 000×), with particles showing typical coronavirus morphology surrounded by dark blue boxes and an individual spike indicated by a red arrow. All samples were stained with 2% (w/v) uranyl acetate. Scale bars represent 100 nm.

To confirm the formation of VLPs, the fraction #6 samples from leaves infiltrated with S alone or EMS were examined by transmission electron microscopy (TEM) using negative staining. The equivalent fraction from EV‐infiltrated leaves was used as negative control (Figure 4). Spiky structures, characteristic of coronavirus VLPs, sized 75–100 nm, were observed in iodixanol‐purified samples from EMS (Figure 4b) and S (Figure 4c,d) while no such structures were observed equivalent in the negative control (Figure 4a). The presence of such higher‐order structures, resembling coronavirus VLPs, indicates that the transiently expressed full‐length S protein can interact with host‐derived membranes to form VLPs. The variation in the size of the VLPs is probably a consequence of the lack of nucleocapsid protein in the preparations, as this acts as a scaffold during virus particle formation. VLPs were also more abundant in the S sample compared to the EMS sample, consistent with the higher levels of S protein expression in the former (Figure 3). The data indicate that the expression of S protein alone is the most efficient approach to generating VLPs. The samples also contained numerous membranous vesicles, presumably of plant origin, since they also occurred in the sample of leaves infiltrated with the EV.

### Furin treatment of SARS‐CoV‐2 VLPs

**Figure 5:**
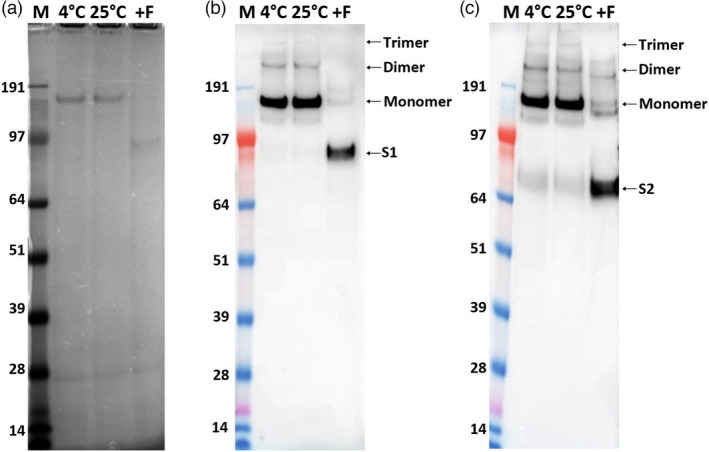
Furin treatment of SARS‐CoV‐2 S protein within VLPs. Purified VLPs were incubated with or without furin and analysed by SDS‐PAGE (a), Western blot using anti‐SARS‐CoV‐2 S protein antibody (b), and SARS‐CoV‐2 Spike S2 subunit antibody (c) after treatment with/without furin. Lane 4 °C, sample incubated at 4 °C without furin; Lane 25 °C, sample incubated at 25 °C without furin; and Lane +F, samples incubated at 25 °C with furin.

To confirm that protein bands are SARS‐CoV‐2, 1 or 2 μg of VLP samples from leaves infiltrated with S alone were incubated with or without furin and the products analysed by SDS–PAGE followed by staining (Figure 5a) or Western blot analysis (Figure 5b,c). Incubation at 25 or 4 °C in the absence of furin did not result in any processing of the full‐length ~140 kDa S protein while incubation with furin resulted in almost total loss of this band and the appearance of lower molecular mass products (Figure 5a). The origin of these was examined by Western blot analysis using either a polyclonal anti‐SARS‐CoV‐2 full‐length S protein antibody or a polyclonal anti‐SARS‐CoV‐2 S2 domain antibody (Figure 5b,c). This analysis identified the products as being the S1 (~90 kDa) and S2 (~70 kDa) domains of the S protein. These sizes were greater than those predicted by the amino acid sequence (https://web.expasy.org/compute_pi/; 75 and 64 kDa, respectively); this is probably a result of glycosylation at least some of the 22 potential N‐glycosylation sites within the S protein. The results indicate that the full‐length S protein within the plant‐made VLPs can be correctly cleaved with furin *in vitro*, supporting the notion the S protein is presented in a biologically relevant conformation.

### Antigenicity test of SARS‐CoV‐2 S proteins and VLPs

**Figure 6:**
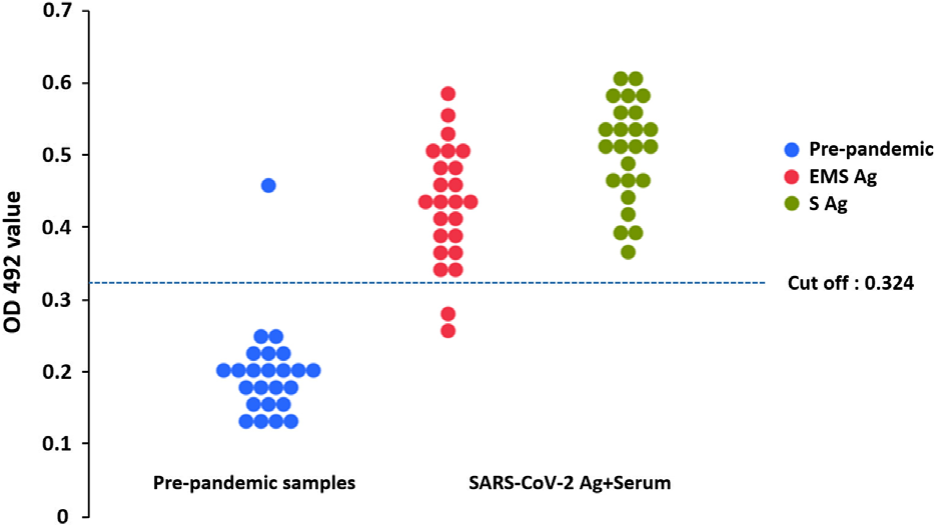
Plant‐derived SARS‐CoV‐2 EMS and S VLPs detect IgG in serum samples from COVID‐19 recovered patients (*n* = 24). The results for EMS and S are indicated by red and green dots, respectively. Results are presented as optical density value (OD) of analysed sera. A cut‐off for positivity was determined as two standard deviations above the mean optical density of pre‐pandemic sera (*n* = 24; blue dots) indicated by a blue dotted line.

To examine whether the S and EMS VLPs could be recognized by human antibodies, we tested human serum from 24 recovering COVID‐19 patients by ELISA. As a negative control, 24 serum samples from individuals collected prior to the pandemic (2017) were analysed in parallel. Using a checkerboard titration, the optimal conditions for the ELISA were determined. Two negative and two anti‐SARS‐CoV‐2 IgG‐positive human sera, as previously determined with commercial chemiluminescent immunoassays (CLIA), were used for a checkerboard titration. The optimal concentration of S protein in the recombinant S and EMS samples was 4 µg/mL, the optimal serum dilution was 1:40, with a secondary antibody dilution of 1:10 000. These conditions produced the highest positive/negative ratio for the standard checkerboard titration (Figure S4). The ELISA assay showed that antibodies produced from recovering COVID‐19 patients can bind plant‐derived S and EMS VLPs, showing the plant‐produced material is immunologically relevant (Figure 6). Serum from a convalescent patient, positive for anti‐SARS‐CoV‐2 IgG, also successfully recognized the recombinant S protein by Western immunoblotting (Figure S5).

### Expression of VLPs containing the S protein of the Delta variant

**Figure 7:**
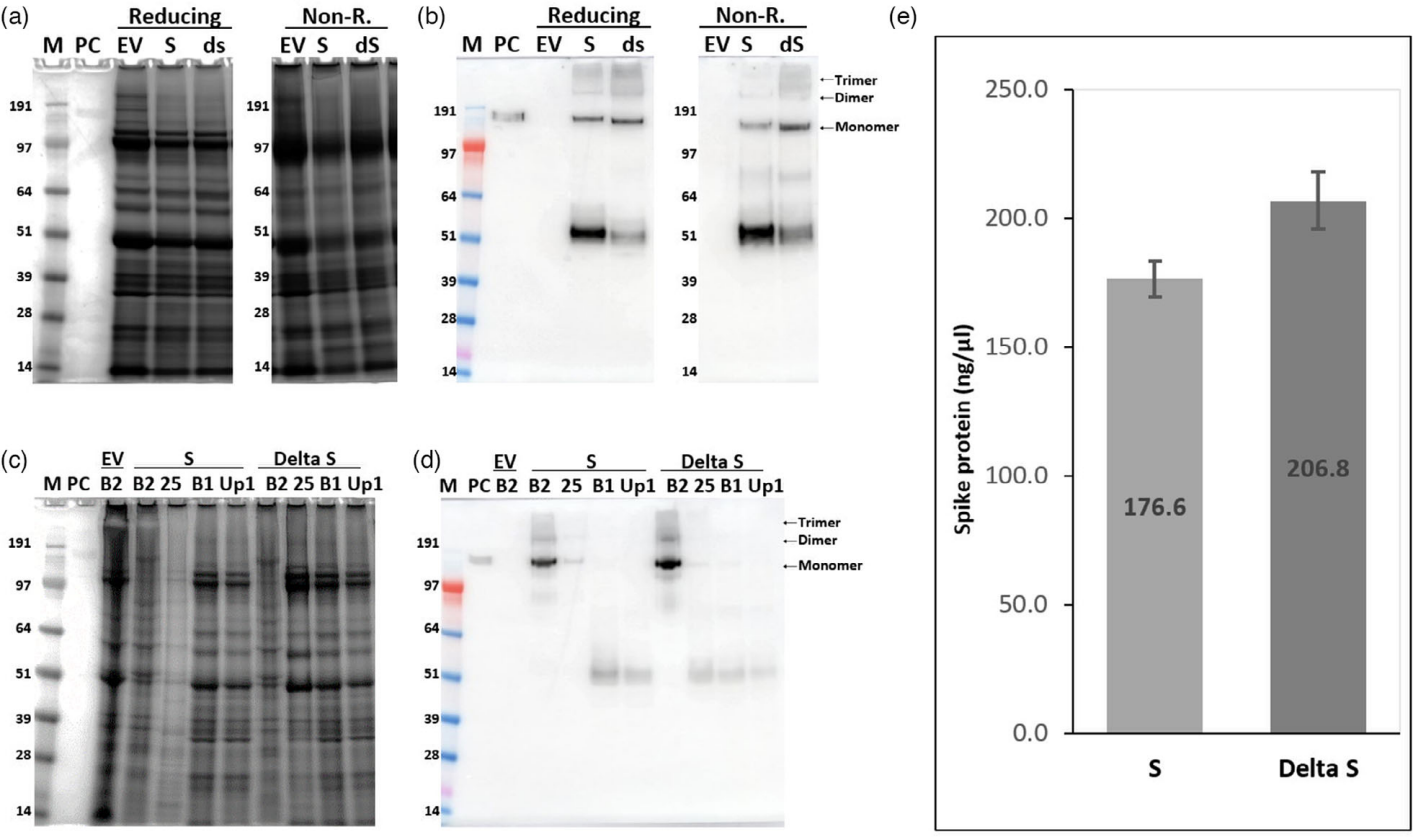
Expression and purification of the S protein of SARS‐CoV‐2 Wuhan strain and Delta variant. SDS–PAGE and Western blot against S protein using protein extracts (a, b) and fractions from sucrose cushions (c, d) of leaves infiltrated with S and Delta S (dS). The S protein concentration in fraction# 6 samples from iodixanol gradients of the S and dS samples was measured by ELISA (e). Error bars indicate the standard deviation of three different wells. Lane M: Protein size marker; Lane PC: 100 ng (for Western blot) or 300 ng (for staining) of SARS‐CoV‐2 spike protein from CHO cells as positive control; reducing: samples boiled in the presence of β‐mercaptoethanol; non‐R: samples boiled in the absence of β‐mercaptoethanol; EV: empty vector controls processed using the same conditions as for S and dS; Lane B2: interface between 25 and 70% sucrose layers plus the 70% layer; Lane 25: 25% sucrose fraction; Lane B1: interface between the supernatant and 25% sucrose fraction; Lane up1: supernatant above the sucrose cushions.

To examine whether the methods developed with the Wuhan strain of SARS‐CoV‐2 can be rapidly deployed to make VLPs containing the S protein of emerging variants, the S protein of SARS‐CoV‐2 Delta variant (dS) was expressed in *N. benthamiana*. The accumulation of dS appeared to be slightly higher than that of the S protein of the original Wuhan strain both in total extracts and in VLPs (Figure 7). Samples of dS showed relatively less of the ~55 kDa‐form (Figure 7b); as with the Wuhan S protein, this form was not present in VLP preparations (Figure 7c,d). After purification on iodixanol gradients (Figure S3c,g), the Wuhan S and dS protein concentrations in the VLP fractions were 176.6 ± 6.9 and 206.8 ± 11.0 ng/µL, respectively (Figure 7e). These levels equate to overall yields of approximatively 24 and 28 mg of full‐length S protein per Kg wet weight mass of infiltrated leaves. This increased yield most likely reflects the decreased level of cleavage of the full‐length S protein found in the case of dS compared to Wuhan S.

## Discussion

The results presented here confirm our preliminary observation (Peyret *et al*., 2021) that it is possible to produce coronavirus VLPs, with a characteristic appearance, in plants by co‐expression of the E, M, and S proteins. However, we have additionally shown that higher levels of S protein and increased yields of VLPs are obtained when the S protein alone is expressed. The higher levels of VLPs seen when the S protein is expressed in the absence of E and M probably relates to the fact that expression of the M protein alone causes necrosis in leaf tissue. This is most likely due to the presence of 3 TM domains on the M protein as the occurrence of such multiple domains on a protein is frequently associated with tissue damage (Thuenemann *et al*., 2013a) and therefore such sequences are often eliminated when producing VLPs of enveloped viruses (Ponndorf *et al*., 2021). Thus, although co‐expression of E and S alleviates the necrosis, it is probable that some damage does occur, limiting expression levels. Due to the lack of antisera specific to E and M we were unable to confirm their incorporation into VLPs when all three proteins were co‐expressed and therefore concentrated mainly on characterizing VLPs produced via the expression of S alone. We have also shown that it is possible to produce and isolate VLPs containing a variant (Delta) of the S protein using the methods developed during this study.

The ability to produce VLPs of SARS‐CoV‐2 by the expression of the S protein alone in plants adds to the findings of Ward *et al*. (2021). These authors showed that a modified form of the S protein, in which the natural leader peptide was replaced by a plant‐derived one, the S1/S2 cleavage site was abolished, mutations were introduced to stabilize the protein in the pre‐fusion form and the TM domain and cytoplasmic tail was replaced with the corresponding sequences from influenza virus, could be incorporated into VLPs. These VLPs have successfully undergone Phase I clinical trials (Ward *et al*., 2021) with the ultimate aim of deploying them as vaccines against SARS‐CoV‐2. By contrast, in the current study, we have used SARS‐CoV‐2 structural proteins with native amino acid sequences with aim of producing VLPs with properties as similar as possible to those of native particles with a view to using them as surrogates for the study of aspects of the virus replication cycle. By producing VLPs containing entirely native versions of the S protein, rather than chimeric molecules, it will be possible to address how amino acid changes between different strains affect such features as stability, glycosylation, and cell attachment. Deliberate modification of the S protein sequence to increase stability and yield, though clearly important for creating candidate vaccines, effectively precludes such comparative analyses as the presence of the stabilizing alterations may well mask subtle differences between strains.

Virus‐like particles, containing full‐length S protein, could be readily separated from truncated forms of the protein by centrifugation through a double sucrose cushion and further purified by centrifugation through iodixanol gradients. This implies that the cleavage products produced in plants are soluble and are either not incorporated into VLPs or are released from them. Thus, the VLPs we have produced contain almost exclusively full‐length S protein and this protein does not undergo cleavage on storage unless exogenous furin is added. This means that the state of the S protein can be modified *in vitro* which will assist the use of such particles in virological studies. The biological relevance of the VLPs produced in plants was demonstrated by the fact that the S protein was recognized by antibodies present in human sera from convalescent patients (Figure 6). The plant‐derived S protein in the S and EMS samples was successfully used as coating antigen to detect anti‐SARS‐CoV2 IgG in serum samples from COVID‐19 recovered patients (Figure 6), which shows that plants produced antigenically relevant protein. In this regard it is worth noting that plant‐produced diagnostic antigens from other viruses have shown high sensitivity and specificity (Siew *et al*., 2021; Takova *et al*., 2021), demonstrating the utility of plant expression systems. The data also suggest that induction of an immune response in the human body might also be possible with the plant‐made VLPs reported here, as previously demonstrated with a modified S protein by Ward *et al*. (2021).

Overall, the results presented here suggest that plants are a suitable expression system for the production of SARS‐CoV‐2 VLPs containing native S protein of both the Wuhan and Delta variants. Alternative strategies to improve VLP purification and scale‐up, such as the use of ultrafiltration, should now be investigated if larger amounts of material are required. Given the ease of expression, and stability of the S protein‐containing VLPs, it should be possible to readily produce VLPs containing further, emerging variants of the S protein. This will facilitate the assessment of the role of mutations in the S protein on such aspects of the antigenicity, stability, and receptor‐binding of the viral particles without the need to handle infectious material.

## Materials and methods

### Vector construction and cloning

The DNA sequences of the S, E and M proteins from SARS‐CoV‐2 isolate Wuhan‐Hu‐1 (NC_045512.2) and Delta variant (MZ359841.1) were codon‐optimized for *N. benthamiana* without amino acid changes and synthesized by GeneArt (Life Technologies Ltd., Renfrew, UK). The sequences were cloned into pEAQ‐*HT* (Sainsbury *et al*., 2009) using AgeI and XhoI restriction sites to give plasmids, pEAQ‐*HT*‐S, pEAQ‐*HT*‐E, and pEAQ‐*HT*‐M, respectively. The DNA was transformed into *Escherichia coli* Top10 cells (Life Technologies Ltd.) and the cloned sequences were confirmed by DNA sequencing. Finally, the plasmids were transformed into *A. tumefaciens* LBA4404 for transient expression in *N. benthamiana*.

### Agroinfiltration

Individual *A. tumefaciens* colonies were propagated in lysogeny broth at 28 °C, 220 rpm for 2 days, and harvested by centrifugation at 4629 ***g*** for 6 min (Sorvall Lynx, ThermoFisher Scientific, Waltham, MA). Pellets from each culture were resuspended in infiltration buffer (10 mm MES, 10 mm MgCl_2_, pH 5.6, 100 μm acetosyringone) and adjusted to an OD_600_ of 0.3 for the expression of the individual proteins, or an OD_600_ of 0.9 and mixed together with 1 : 1 : 1 ratio (final OD_600_ of each Agrobacterium suspension is 0.3) for the co‐infiltration of all three constructs. *N. benthamiana* plants were grown on custom‐mixed soil comprising of peat, 2.5 kg/m^3^ dolomite limestone, 1.3 kg/m^3^ base fertilizer, 2.7 kg/m^3^ Osmocote^®^ (applied every 3–4 months), 0.3 kg/m^3^ Exemptor^®^, and 0.25 kg/m^3^ wetter in a controlled environment of 16‐h photoperiod generated by 400 W sodium lamps, 24 °C and 70% relative humidity (Pang *et al*., 2019). The first three mature leaves of plants grown for 3 weeks after pricking out were infiltrated using a needless syringe (Thuenemann *et al*., 2013a, 2013b) and maintained at 23–25 °C with 16 h lighting.

### Protein extraction and purification

Infiltrated leaves were harvested at 6 dpi, weighed and blended in three volumes of TEN buffer (10 mm Tris–HCl, 1 mm EDTA, 1 m NaCl, pH 7.4). To assess the level of S protein expression, a total of 6 leaf discs were punched from the infiltrated regions using a cork borer (number 6, Merck, Sigma–Aldrich Co. Ltd., Burlington, MA, catalogue number Z165220) and homogenized in 270 µL TEN buffer using a Bead Ruptor 24 (Camlab, Cambridge, UK), speed = 4, 30 s, 4 °C). Homogenized samples were centrifuged (10 min, 16 000 ***g***, 5 °C), the supernatant was collected, and the extraction was repeated under the same conditions. For scaled up extraction, 15 g of infiltrated leaves were blended with 45 mL of TEN buffer and the homogenized samples were filtered through a double layer of Miracloth (Merck Millipore, Burlington, MA) and centrifuged at 1575 ***g*** for 30 mins at 5 °C using a Sorvall Lynx 4000 centrifuge (Thermo Fisher Scientific, Waltham, MA). The supernatant was filtered through a 0.45 μm syringe filter (Merck Millipore, Sartorius, Burlington, MA) and 31.5 mL of each sample was loaded onto a double layer sucrose cushion [5 mL of 25% (w/v) and 1 mL of 70% (w/v) sucrose prepared in PBS (pH 7.0)], followed by centrifugation for 3 h at 167 000 ***g*** (30 000 rpm in a SureSpin 360/36 rotor, Thermo Fisher Scientific). Two milliliter fractions were collected from the bottom of the tube (70% and interface between 70% and 25% sucrose, 25% sucrose, and 2 mL of supernatant) and used for Western blot analysis. Fractions from the 70% sucrose layer and the interface were desalted through PD‐10 desalting column (17085101, GE Healthcare, Chicago, IL), and filtered through 0.22 μm syringe filters (Merck Millipore, Satorious), and then 1.8 ml of samples were loaded onto 12%–30% iodixanol gradients [2.5 mL each of 12%, 18%, 24%, 30% (v/v), OptiPrep™ density gradient medium, (Merck Sigma)]. A 1.5 mL sample of supernatant and 1 mL fractions starting from the interface between supernatant and 12% were collected after centrifugation for 2.5 h at 4 °C, 260 000 ***g*** in a TH641 rotor, Sorvall). Each fraction was analysed by Western blot using anti‐SARS‐CoV‐2 S protein antibody and fractions showing bands corresponding in size to the S protein were desalted through PD‐10 columns 7085101 (GE Healthcare) and used for further analysis.

### SDS–PAGE and Western blot analysis

Samples were denatured in LDS sample buffer with or without beta‐mercaptoethanol and boiled for 5 min and electrophoresed through 4%–12% (w/v) NuPAGE Bis‐Tris gels (Novex, Thermo Fisher Scientific). The proteins were either transferred to nitrocellulose membranes or stained with InstantBlue^®^ (Abcam, Cambridge, UK). SARS‐CoV‐2 S glycoprotein (Trimeric), His‐Tag from CHO cells (REC31871, The Native Antigen Company, Oxford, UK) was used as a positive control. Membranes were blocked in 10% (w/v) skim milk in Tris–buffered saline with Tween‐20 (0.1% v/v; TBST) on a rotary shaker overnight. S protein was detected using a 1 : 2000 dilution of rabbit anti‐SARS‐CoV‐2 S protein polyclonal antibody (MBS434243, MyBioSource, San Diego, CA), followed by a 1 : 7000 dilution of anti‐rabbit IgG horseradish peroxidase (HRP) (ab190584, Abcam) as the secondary antibody. S2 subunit was detected by 1:500 dilution of SARS‐CoV‐2 S2 Subunit Antibody (MAB10557, R&D Systems, Minneapolis, MN), followed by 1 : 7000 dilution of Goat anti‐Mouse IgG (H + L) Secondary Antibody, HRP (62‐6520, Thermo Fisher Scientific). Chemiluminescence was detected using an ImageQuant LAS 500 (GE Healthcare).

For Western blot analysis using human serum from a convalescent patient, the proteins were transferred from the 10% (w/v) SDS–PAGE gel onto a polyvinylidene difluoride membrane. The membrane was blocked with 5% (w/v) nonfat dried milk and 1% (w/v) bovine serum albumin (BSA) in TBST for 1 h. The membrane was incubated overnight at 4 °C with a human serum positive for anti‐S Ag [diluted 1 : 75 in TBST with 3% (w/v) BSA] and washed four times with TBST. The bound antibody was detected with secondary Goat F(ab')2 Anti‐Human IgG‐Fc (AP), (ab98588, Abcam) diluted 1 : 10 000 in blocking buffer. One‐step NBT/BCIP substrate (Thermo Fisher Scientific) was used.

### S protein quantification by ELISA

Fifty microlitres samples of fraction #6 from the iodixanol gradients of the EV, EMS, S, and dS samples were loaded into wells of 96 well plates that contained 50 µL PBS and the samples serially diluted 1 : 2. The plates were incubated at 4 °C overnight to allow protein coating. SARS‐CoV‐2 S glycoprotein (Trimeric), His‐Tag from CHO cells (REC31871, The Native Antigen Company) was used to create a standard curve. Plates were washed with PBS containing 0.05% (v/v) Tween‐20 (PBS‐T) three times and blocked using 200 µL/well of 1% (w/v) BSA in PBS‐T and incubated at 25 °C for 2 h, followed by three washes with PBS‐T. The wells were loaded with 100 μL/well of a 1 : 2000 dilution of rabbit anti‐SARS‐CoV‐2 S protein polyclonal antibody (MBS434243, MyBioSource) in 1% (w/v) BSA in PBS‐T and incubated for 1 h at room temperature, followed by three washes with PBS‐T. The plate was then incubated with 100 μL/well of a 1 : 7000 dilution of the secondary antibody (anti‐rabbit IgG conjugated with HRP; ab190584, Abcam), for 1 h at room temperature in the dark, and washed four times with PBS‐T. Plates were finally incubated for 20 min at room temperature with 100 μL/well of TMB substrate (34021, Thermo Fisher Scientific). The reaction was stopped by adding sulphuric acid to a final concentration of 1 m. The optical density was measured at 450 nm using a multi‐mode microplate reader (FLUOstar Omega, BMG Labtech, Ortenberg, Germany). The R‐squared values of the standard curve from each experiment ranged from 0.99 to 1.0; samples with OD_450nm_ values outside the range of the standard curve were not included. Standard deviations from two (for dS) or three (for S) independent experiments were used to generate error bars.

### Furin treatment

Cleavage of the S protein into S1 and S2 was assessed by treatment with human furin (F2677, Sigma–Aldrich) according to the manufacturer’s instructions with slight modification. Briefly, 1 or 2 μg of purified S protein based on ELISA data within the VLP fraction was incubated with/without furin for 24 h at 25 °C and compared with samples incubated at 4 °C without any treatment by SDS–PAGE. Western blot analysis was carried out using an anti‐SARS‐CoV‐2 S protein polyclonal antibody or a SARS‐CoV‐2 S2 subunit antibody.

### Serum sample collection

Samples were collected from SARS‐CoV‐2 infected volunteers (*n* = 24) from 4 April until 1 July 2021. Samples were taken after the full recovery of the patients who had had moderate COVID‐19 illness. A panel of 24 human serum from COVID‐19 recovered patents and 24 pre‐pandemic serum samples from patients, was used to determine if plant‐produced S and EMS VLPs were able to recognize SARS‐CoV‐2 specific antibodies. Ethical approval for this study was obtained by the IMBB institutional ethic committee (process number EK‐19042021). All participants provided written informed consent.

### ELISA using human sera

Wells were coated with 2, 4, or 8 µg/mL of the S VLPs or 2 or 4 µg/mL of the EMS VLPs in bicarbonate/carbonate coating buffer (100 mm), pH 9.6. The microtiter plates (Greiner 96‐well flat bottom) were coated with 50 µL/well of purified coating protein and incubated overnight at 4 °C. After three washes with PBST, the plates were incubated with 200 µL/well of blocking solution [3% (w/v) BSA in PBST] for 1 h at room temperature. Aliquots of sera diluted 1 : 20; 1 : 40; 1 : 80 in blocking buffer [1% (w/v) BSA in PBST] repeated in duplicates were dispensed into the wells of the plates and incubated for one hour at 37 °C. After three washes with PBST, anti‐human IgG (H + L) antibody, peroxidase‐labelled (KPL, LGS Sera Care, Milford, MA) was added at a dilution of 1 : 5000; 1 : 10 000, or 1 : 20 000. After incubation with the secondary antibody, wells were washed three times before 50 µL/well of the substrate solution (o‐phenylenediamine, Millipore‐Sigma, Munich, Germany) was added. Plates were incubated in the dark at room temperature for 20 min. The reaction was stopped by adding sulphuric acid to a final concentration of 1 m and the plates were read at 492 nm in an Epoch Microplate Spectrophotometer plate reader (BioTek Instruments Inc., Winooski, VT). The mean positive/negative (P/N) ratio was calculated. Two negative and two positive human sera previously determined with CLIA test LIAISON^®^ SARS‐CoV‐2 IgG kit (Diasorin, Saluggia, Italy) were used for a checkerboard titration. The optimal concentration of recombinant S and EMS VLPs used to coat the ELISA plate was 4 µg/mL using a 1 : 40 dilution of the serum and 1 : 10 000 secondary antibody dilution. This produced the highest positive/negative ratio for the standard checkerboard titration.

### Transmission electron microscopy

Samples were applied to 400‐mesh carbon‐coated copper grids (EM Resolution, Sheffield, UK) and incubated for 30 s. The grids were washed with distilled water 3 times and stained with 2% (w/v) uranyl acetate for 30 s and imaged using Talos F200C electron microscope (Thermo Fisher Scientific).

### Accession numbers

The *N. benthamiana* codon‐optimized versions of SARS‐CoV‐2 genes within the pEAQ‐*HT* vectors used in this work have the following accession numbers:

Isolate Wuhan‐Hu‐1 E protein: OK413876
Isolate Wuhan‐Hu‐1 M protein OK413877
Isolate Wuhan‐Hu‐1 S protein OK413878
Delta variant S protein: OM858819

## Conflict of interest

G.P.L. declares that he is a named inventor on granted patent WO 29087391 A1 which describes the HyperTrans expression system and associated pEAQ vectors used in this manuscript.

## Author contributions

Conceptualization, J.‐W.J. and G.P.L.; methodology, J.‐W.J. and G.Z.; validation, all authors; formal analysis, all authors; investigation, J.‐W.J. and G.Z.; resources, G.P.L. and I.M.; data curation, J.‐W.J. and G.Z.; writing—original draft preparation, J.‐W.J, G.P.L., and G.Z.; writing—review and editing, all authors; supervision, G.P.L. and I.M.; project administration, G.P.L. and I.M.; and funding acquisition, J.‐W.J., G.P.L., and I.M. All authors have read and agreed to the published version of the manuscript.

## Supporting information


**Figure S1** Effect of the expression of SARS‐CoV‐2 proteins on N.benthamiana leaves. Leaves were infiltrated with either individual Agrobacterium suspensions harbouring the S, M and E proteins or a combination of all three and the leaves photographed at either 4 (a) or 6 (b) DPI.
**Figure S2** Behaviour of degraded forms of S protein during centrifugation through sucrose cushions.
**Figure S3** Further purification of VLPs from leaves infiltrated with S, EMS or dS using iodixanol gradients. Each B2 fraction from sucrose cushions of S, EMS and dS samples were desalted using PD‐10 column and loaded onto iodixanol gradients (12, 18, 24 and 30%).
**Figure S4** Determination of optimal in‐house ELISA conditions (antigen coating concentration and serum dilution). Two positive and two negative serum samples previously determined with commercial CLIA kit were used in duplicates.
**Figure S5** Determination of binding of S to convalescent serum by the western blot. Lane 1, purified fraction #6 from empty vector infiltrated leaves; Lane 2, Crude extract from EV leaves; Lane 3, purified fraction #6 from S infiltrated leaves.
