# Supplementary material for: Plant‐based expression and characterization of SARS‐CoV‐2 virus‐like particles presenting a native spike protein
Source: Plant Biotechnol J. 2022 Apr 6;20(7):1363–72. doi: 10.1111/pbi.13813 (PMC9115404; doi:10.1111/pbi.13813)
Supplement: Supplementary file 1 — Figure S1 Effect of the expression of SARS‐CoV‐2 proteins on N.benthamiana leaves. Leaves were infiltrated with either individual Agrobacterium suspensions harbouring the S, M and E proteins or a combination of all three and the leaves photographed at either 4 (a) or 6 (b) DPI. Figure S2 Behaviour of degraded forms of S protein during centrifugation through sucrose cushions. Figure S3 Further purification of VLPs from leaves infiltrated with S, EMS or dS using iodixanol gradients. Each B2 fraction from sucrose cushions of S, EMS and dS samples were desalted using PD‐10 column and loaded onto iodixanol gradients (12, 18, 24 and 30%). Figure S4 Determination of optimal in‐house ELISA conditions (antigen coating concentration and serum dilution). Two positive and two negative serum samples previously determined with commercial CLIA kit were used in duplicates. Figure S5 Determination of binding of S to convalescent serum by the western blot. Lane 1, purified fraction #6 from empty vector infiltrated leaves; Lane 2, Crude extract from EV leaves; Lane 3, purified fraction #6 from S infiltrated leaves. [file PBI-20-1363-s001.pptx]

## Slide 1
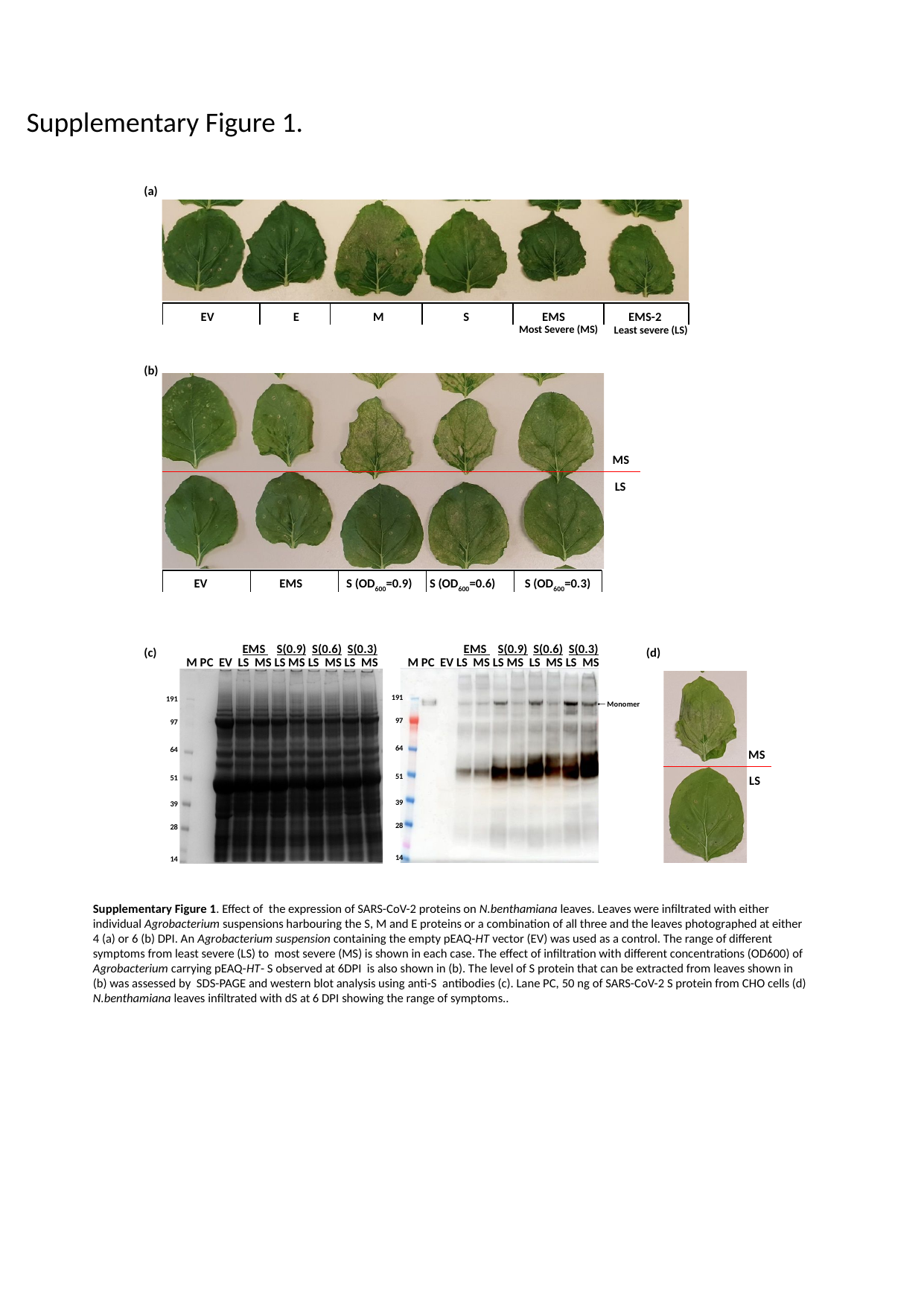

Supplementary Figure 1.
(a)
EV
E
M
S
EMS
EMS-2
Most Severe (MS)
Least severe (LS)
(b)
MS
LS
EV
EMS
S (OD600=0.9)
S (OD600=0.6)
S (OD600=0.3)
 EMS S(0.9) S(0.6) S(0.3)
 EMS S(0.9) S(0.6) S(0.3)
(c)
(d)
M PC EV LS MS LS MS LS MS LS MS
M PC EV LS MS LS MS LS MS LS MS
191
191
Monomer
97
97
64
64
MS
51
LS
51
39
39
28
28
14
14
Supplementary Figure 1. Effect of the expression of SARS-CoV-2 proteins on N.benthamiana leaves. Leaves were infiltrated with either individual Agrobacterium suspensions harbouring the S, M and E proteins or a combination of all three and the leaves photographed at either 4 (a) or 6 (b) DPI. An Agrobacterium suspension containing the empty pEAQ-HT vector (EV) was used as a control. The range of different symptoms from least severe (LS) to most severe (MS) is shown in each case. The effect of infiltration with different concentrations (OD600) of Agrobacterium carrying pEAQ-HT- S observed at 6DPI is also shown in (b). The level of S protein that can be extracted from leaves shown in (b) was assessed by SDS-PAGE and western blot analysis using anti-S antibodies (c). Lane PC, 50 ng of SARS-CoV-2 S protein from CHO cells (d) N.benthamiana leaves infiltrated with dS at 6 DPI showing the range of symptoms..

## Slide 2
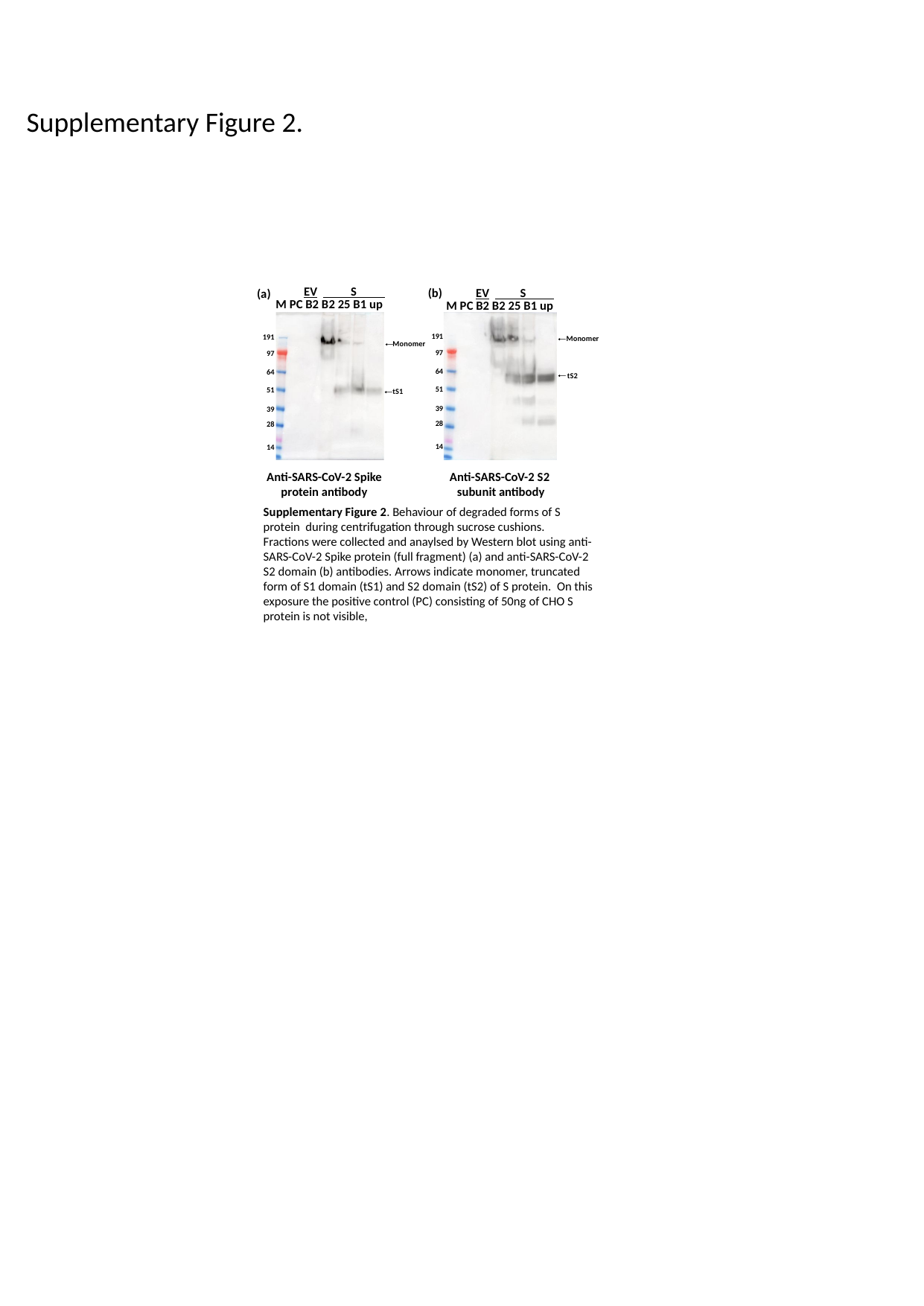

Supplementary Figure 2.
 EV S .
EV S .
(b)
(a)
M PC B2 B2 25 B1 up
M PC B2 B2 25 B1 up
191
191
Monomer
Monomer
97
97
64
64
tS2
51
51
tS1
39
39
28
28
14
14
Anti-SARS-CoV-2 Spike protein antibody
Anti-SARS-CoV-2 S2
subunit antibody
Supplementary Figure 2. Behaviour of degraded forms of S protein during centrifugation through sucrose cushions. Fractions were collected and anaylsed by Western blot using anti-SARS-CoV-2 Spike protein (full fragment) (a) and anti-SARS-CoV-2 S2 domain (b) antibodies. Arrows indicate monomer, truncated form of S1 domain (tS1) and S2 domain (tS2) of S protein. On this exposure the positive control (PC) consisting of 50ng of CHO S protein is not visible,

## Slide 3
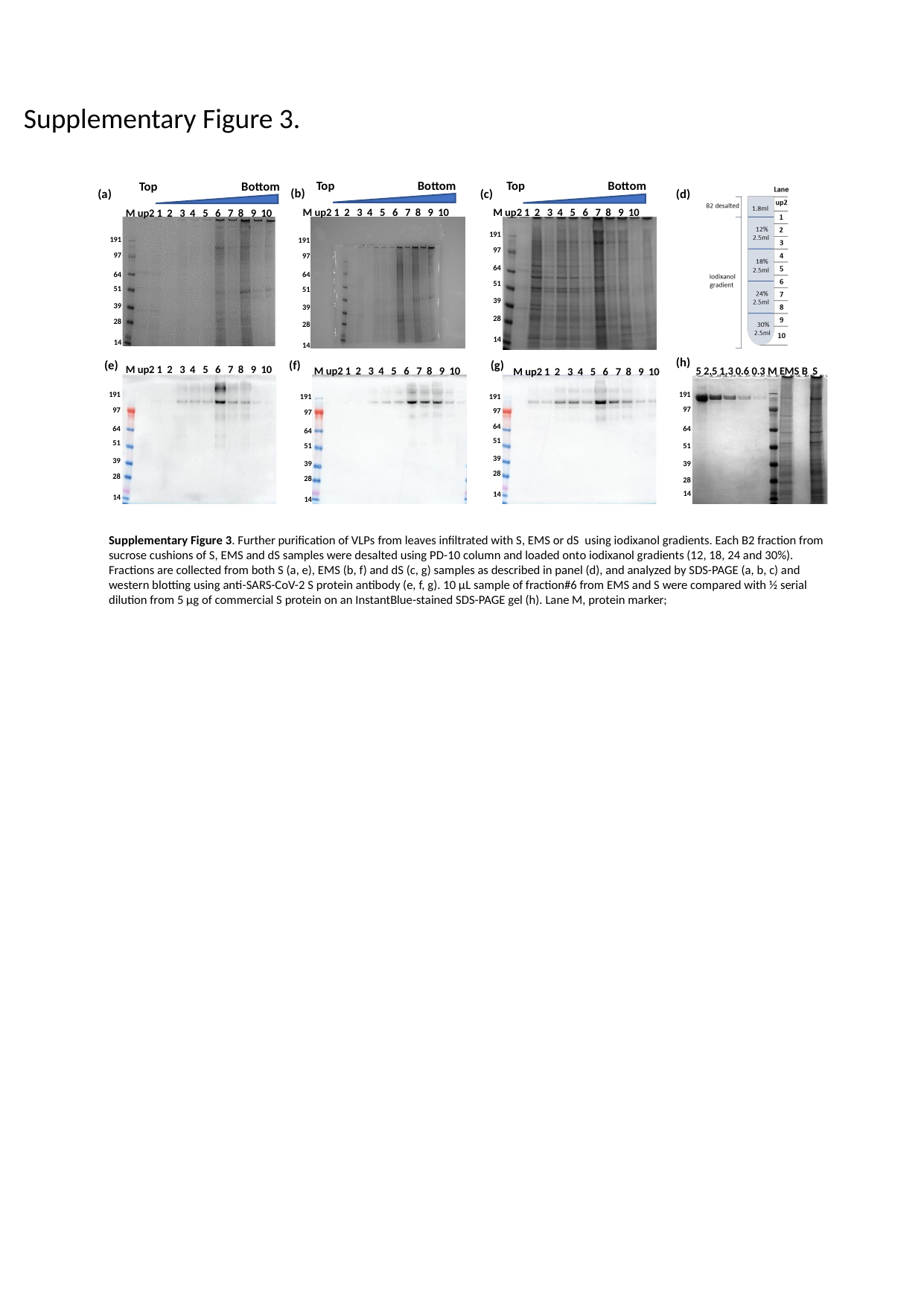

Supplementary Figure 3.
Top
Bottom
Top
Bottom
Top
Bottom
(b)
(c)
(a)
(d)
M up2 1 2 3 4 5 6 7 8 9 10
M up2 1 2 3 4 5 6 7 8 9 10
M up2 1 2 3 4 5 6 7 8 9 10
191
191
191
97
97
97
64
64
64
51
51
51
39
39
39
28
28
28
14
14
14
(h)
(f)
(g)
(e)
M up2 1 2 3 4 5 6 7 8 9 10
M up2 1 2 3 4 5 6 7 8 9 10
 5 2.5 1.3 0.6 0.3 M EMS B S
M up2 1 2 3 4 5 6 7 8 9 10
191
191
191
191
97
97
97
97
64
64
64
64
51
51
51
51
39
39
39
39
28
28
28
28
14
14
14
14
Supplementary Figure 3. Further purification of VLPs from leaves infiltrated with S, EMS or dS using iodixanol gradients. Each B2 fraction from sucrose cushions of S, EMS and dS samples were desalted using PD-10 column and loaded onto iodixanol gradients (12, 18, 24 and 30%). Fractions are collected from both S (a, e), EMS (b, f) and dS (c, g) samples as described in panel (d), and analyzed by SDS-PAGE (a, b, c) and western blotting using anti-SARS-CoV-2 S protein antibody (e, f, g). 10 µL sample of fraction#6 from EMS and S were compared with ½ serial dilution from 5 µg of commercial S protein on an InstantBlue-stained SDS-PAGE gel (h). Lane M, protein marker;

## Slide 4
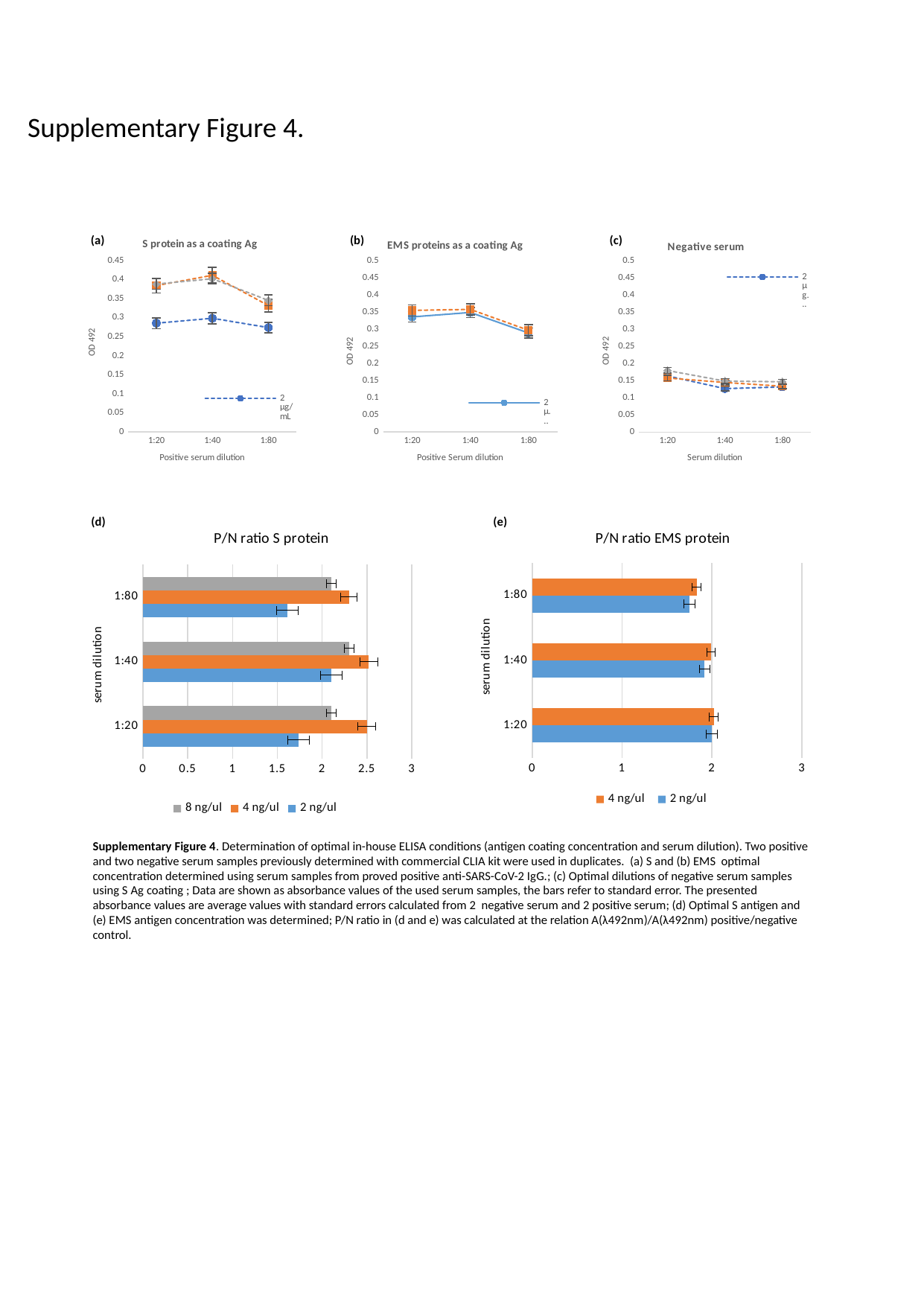

Supplementary Figure 4.
(b)
(c)
### Chart: S protein as a coating Ag
| Category | 2 µg/mL | 4 µg/mL | 8 µg/mL |
|---|---|---|---|
| 1:20 | 0.286 | 0.385 | 0.389 |
| 1:40 | 0.299 | 0.412 | 0.403 |
| 1:80 | 0.2745 | 0.3324 | 0.346 |(a)
### Chart: EMS proteins as a coating Ag
| Category | 2 µg/mL | 4 µg/mL | | |
|---|---|---|---|---|
| 1:20 | 0.337 | 0.356 | None | None |
| 1:40 | 0.35 | 0.359 | None | None |
| 1:80 | 0.29 | 0.298 | None | None |
### Chart: Negative serum
| Category | 2 µg/mL | 4 µg/mL | 8 µg/mL |
|---|---|---|---|
| 1:20 | 0.164 | 0.158 | 0.18 |
| 1:40 | 0.127 | 0.145 | 0.149 |
| 1:80 | 0.132 | 0.134 | 0.147 |
### Chart: P/N ratio EMS protein
| Category | 2 ng/ul | 4 ng/ul |
|---|---|---|
| 1:20 | 2.0 | 2.02 |
| 1:40 | 1.92 | 1.99 |
| 1:80 | 1.75 | 1.83 |(e)
(d)
### Chart: P/N ratio S protein
| Category | 2 ng/ul | 4 ng/ul | 8 ng/ul |
|---|---|---|---|
| 1:20 | 1.74 | 2.5 | 2.1 |
| 1:40 | 2.1 | 2.52 | 2.3 |
| 1:80 | 1.61 | 2.3 | 2.1 |Supplementary Figure 4. Determination of optimal in-house ELISA conditions (antigen coating concentration and serum dilution). Two positive and two negative serum samples previously determined with commercial CLIA kit were used in duplicates. (a) S and (b) EMS optimal concentration determined using serum samples from proved positive anti-SARS-CoV-2 IgG.; (c) Optimal dilutions of negative serum samples using S Ag coating ; Data are shown as absorbance values of the used serum samples, the bars refer to standard error. The presented absorbance values are average values with standard errors calculated from 2 negative serum and 2 positive serum; (d) Optimal S antigen and (e) EMS antigen concentration was determined; P/N ratio in (d and e) was calculated at the relation A(λ492nm)/A(λ492nm) positive/negative control.

## Slide 5
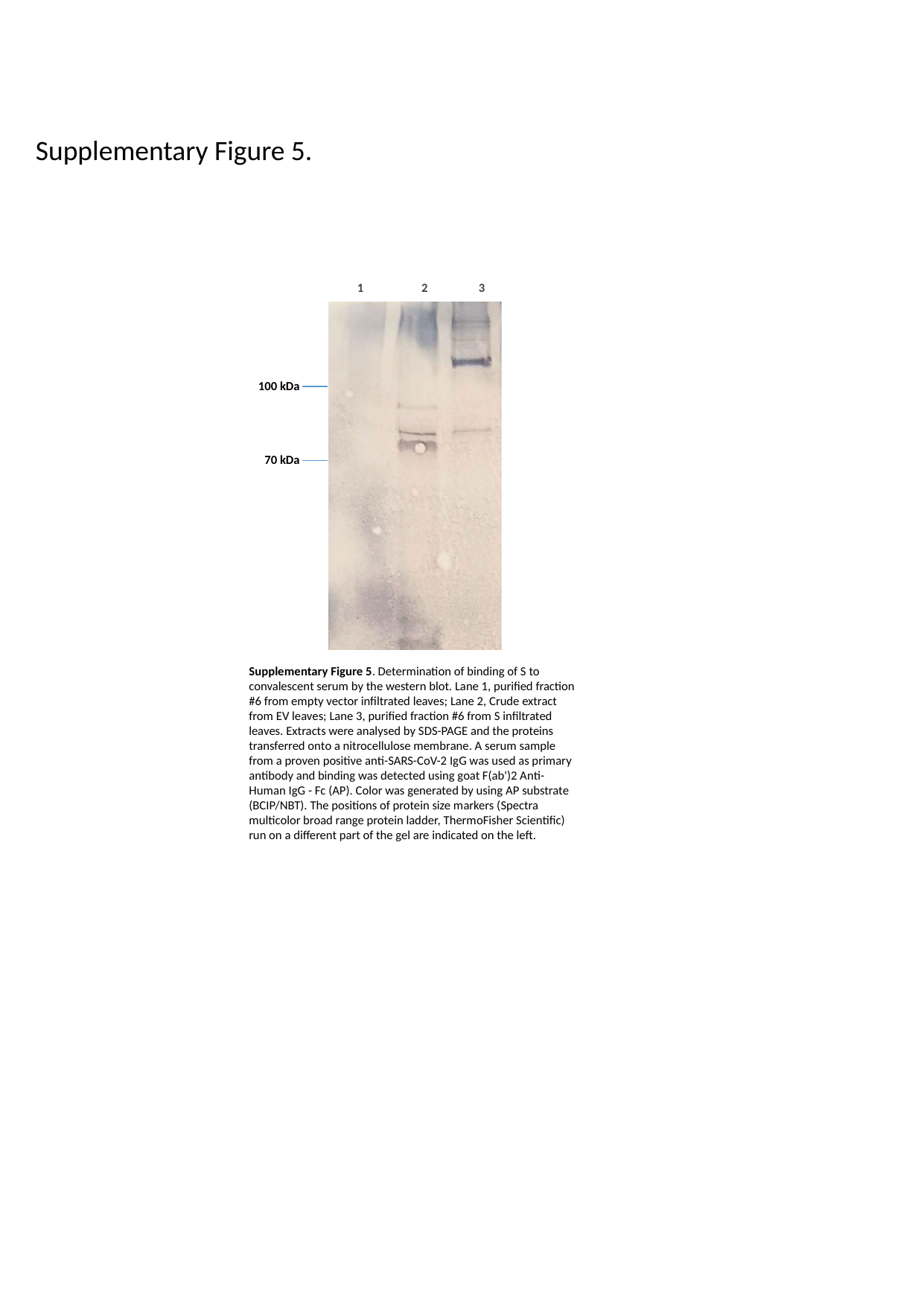

Supplementary Figure 5.
1 2 3
100 kDa
70 kDa
Supplementary Figure 5. Determination of binding of S to convalescent serum by the western blot. Lane 1, purified fraction #6 from empty vector infiltrated leaves; Lane 2, Crude extract from EV leaves; Lane 3, purified fraction #6 from S infiltrated leaves. Extracts were analysed by SDS-PAGE and the proteins transferred onto a nitrocellulose membrane. A serum sample from a proven positive anti-SARS-CoV-2 IgG was used as primary antibody and binding was detected using goat F(ab')2 Anti-Human IgG - Fc (AP). Color was generated by using AP substrate (BCIP/NBT). The positions of protein size markers (Spectra multicolor broad range protein ladder, ThermoFisher Scientific) run on a different part of the gel are indicated on the left.
